# In vitro study on the impact of various polishing systems and coffee staining on the color stability of bleach-shaded resin composite

**DOI:** 10.1186/s12903-024-04474-5

**Published:** 2024-06-20

**Authors:** Ali Jrady, Hala Ragab, Fahda N. Algahtani, Essam Osman

**Affiliations:** 1 Beirut Arab University, Beirut, Lebanon; 2 Operative and Esthetic Dentistry, Clinical Dental Sciences, College of Dentistry, Princess Nourah Bint Abdul Rahman University, P.O. Box 84428, 11671 Riyadh, Saudi Arabia; 3 Clinical Dental Sciences, College of Dentistry, Princess Nourah Bint Abdul Rahman University, P.O. Box 84428, 11671 Riyadh, Saudi Arabia; 4 Dental BioMaterials, Faculty of Dentistry, Alexandria University, Alexandria, Egypt

**Keywords:** Bleach-shade composite, Nanohybrid composite, Microhybrid composite, Coffee stain, And finishing and polishing

## Abstract

**Objective:**

To evaluate the effects of different polishing techniques and coffee staining on the color stability of four commercially available bleach-shade composite resins, namely microhybrid, nanohybrid, nanofilled, and injectable nanohybrids.

**Material and methods:**

A total of 224 discs (8 mm diameter and 2 mm thickness) were fabricated from four different bleach-shade composite resins, namely microhybrid (Gradia Direct Anterior), nanohybrid (Palfique LX5), nanofilled (Filtek Universal), and injectable nanohybrid (flowable G-aenial universal injectable). The composite resin groups were polished via four techniques: no polishing, 4-step polishing using aluminum oxide discs, 3-step polishing using silicon rubber diamond discs, and one-step polishing. Half of each group was immersed in water, while the other half was immersed in coffee for 12 days (*n* = 7). Colors were measured using a clinical spectrophotometer, and color differences were calculated (ΔE). The results were analyzed statistically.

**Results:**

The alterations in color were significantly influenced by the techniques employed for finishing and polishing techniques, composite resin type, and degree of coffee staining. Regardless of the polishing technique and storage medium, different material types showed a significant color change (ΔE) at *P* < 0.001. Filtek exhibited the most significant color change, followed by Gradia and Palfique, with no significant differences between them. In addition, Different polishing techniques resulted in significant color changes (*P* < 0.001). The highest degree of color change was seen in the no-polishing group, followed by the 4-step and 1-step polishing groups, with negligible differences between each other. Also, Storage media had a significant effect on ΔE values.

**Conclusion:**

Appropriate finishing and polishing procedures can improve the color stability of bleach-shaded composite resins. Coffee has a deleterious effect on color; however, injectable flowable nanohybrid composites are more resistant to staining.

## Clinical relevance

The study provides additional information on the color stability of bleach-shaded composite resins post-polishing and coffee staining, highlighting the importance of understanding the material type, polishing protocol, and coffee consumption in determining the longevity and esthetic outcome of these restorations. Current knowledge on bleach-shade composite resins is limited, highlighting the need for extensive research and further clinical studies.

## Introduction

In recent years, under the influence of social media, people have become obsessed with perfectly aligned white teeth. Consequently, the number of patients seeking smile makeovers with bright white veneers has increased significantly. Because of its economic advantages over porcelain veneers, direct bonding with a composite resin has become a popular choice [1].

A variety of composite resins with bleach-shades and varying compositions have been launched in the market, including nanofills, nano- and microhybrids, and the recently introduced injectable flowable nanohybrid composite resins. Microhybrid composites, with particle size averages of 0.4–1.0 μm, have high strengths. Nanohybrid composite resins, which contain pre-polymerized resin fillers and nanoclusters and combine nanometer-sized particles with conventional filler technology, are known for their remarkable esthetic properties [1]. Nanofilled composites utilize nanosized particles, ranging from 5–20 nm in the form of discrete or fused nanoclusters with an average size 0.6–10 µm, throughout the resin matrix. Nanohybrid injectable composites have demonstrated their good handling and optical properties via a monoshade restorative procedure [2].

Because of their "chameleon effect" characteristics, composite resins with nanofillers are expected to offer superior esthetic qualities and improved color harmony with dental tissues [3].

The main difference between the bleach-shaded composite resin and other shades was the type of photoinitiator used. Unlike the regular shades of composite resins that utilize type II photoinitiators (camphorquinone CQ), bleach-shaded composite resins utilize type I white and colorless photoinitiators such as lucirin TPO and phenylpropanedione, either alone or in combination with CQ, to avoid the photoyellowing effect of the composite [4].

Despite recent advances in the material science of composite resins, the color stability in bleach and other shades of composites resins remains a significant challenge [5]. Both internal and external factors can contribute to color changes. Extrinsic factors typically include poor oral hygiene, smoking, and the consumption of colored foods and drinks. Numerous studies have reported varying levels of discoloration in composite resin restorations caused by beverages such as coffee, tea, cola, and red wine [6, 7]. Coffee was reported to have higher potential for the discoloration of nanofilled and microhybrid composites than tea and Coca-Cola [8]. Coffee affects the surface roughness of composite resins more than distilled water and Coca-Cola [9]. Intrinsic causes are related to the chemical composition of composite resins [10]. Because intrinsic factors are totally dependent on the formulation of the manufacturer, dental practitioners cannot prevent intrinsic color changes but can minimize them by ensuring adequate polymerization, proper finishing, polishing, and regular maintenance [10].

Color stability of the dental restorations was assessed using both visual and instrumental techniques. Instruments such as colorimeters, spectrophotometers, and digital cameras were utilized to measure the color changes (ΔE*) by referring to the standards of the Commission Internationale de L’éclairage (CIE) system [11]*.* Different thresholds of ΔE values have been reported in various studies, beyond which the color difference become visual to the human eye. According to Paravina et al. (2019), the acceptability of color difference for CIELAB color coordinate (ΔE) should be less than or equal to 2.7 [12].

A smooth surface reduces the risk of discoloration; therefore, several finishing and polishing techniques have been used to smoothen the surface of composite resin restorations [13]. The smoothest surface is produced when the composite resin is polymerized via a strip matrix; yet, the least color stable due to oxidation. Many essential clinical steps such as excess material removal and restoration recontouring alter the results produced by the strip matrix [14]. Contemporary finishing and polishing techniques involve the use of diamond and carbide finishing burs, ceramic diamond rotary instruments with hard-bonded/surface-coated ceramic diamonds, aluminum oxide-impregnated rubber or silicon discs, and wheels. The outcome of polishing technique can vary depending on the abrasive grit, particle size, and hardness [15]. Moreover, the type of dental composite used can influence the surface roughness, because the type of filler particles and hardness of the resin matrix are crucial factors affecting the surface roughness [4]. Therefore, it is important to investigate the factors that could influence the surface roughness and color stability of different types of composites designed to be placed in the esthetic zone. Despite extensive research on the impact of various polishing and finishing techniques on the color stability of A2- and A1-shaded composite resins, the color stability of bleach-shaded composite resins remains largely unexplored in the literature.

This study aims to evaluate the effects of different polishing techniques and coffee storage on the color stability of four different types of bleach-shade composites, namely microhybrid, nanohybrid nanofilled, and injectable nanohybrid. The null hypothesis was that different polishing techniques and coffee staining would not affect the color stability of the tested bleach-composite resins.

## Materials & methods

In this study, the color changes of four types of bleach-shade composite resins, namely microhybrid (Gradia direct anterior), nanohybrid-flowable (G-aenial universal injectable), nanohybrid (Palifique Lx5), and nanofilled (Filtek Universal) (Table 1), were examined after applying different polishing techniques (Table 2).

**Table 1:** Materials description

Materials-shade	Type	Composition	Filler size-wt/vol	Manufacturer- Batch number
Gradia Direct Anterior Shade XBW	Microhybrid composite	UDMA dimethacrylate trimethacrylate fluoro-alumino-silicate glass silica powder	0.85 microns average size/ 73%wt 64% vol	GC corporation (Tokyo, Japan) 190914A
G-aenial Universal Injectable Shade BW	Flowable- Nanohybrid composite	Bis-MEPP (Bis-EMA), UDMA, TEGDMA Silicon dioxide strontium glass	10–200 nm/ 69% wt 50% vol	GC corporation (Tokyo, Japan) 210209B
Palfique LX5 Shade SW	Nanohybrid composite	Bis-GMA, TEGDMA Silica-zirconia filler Composite filler	0.1–0.3µm/ 82% wt 71% vol	Tokoyama Dental Corporation Inc (Japan) 05E21
Filtek Universal Shade XW	Nanofill composite	AUDMA, AFM diurethane-DMA, 1,12-dodecane-DMA Silica(20nm), zirconia particles(4-11nm), ytterbium trifluoride (100nm)	4-20nm non-aggregated 100nm aggregated/ 76.5% wt 58.4% vol	3 M ESPE (St. Paul, MN, USA

**Table 2:** Finishing and polishing systems

System	Description	Manufacturer-batch number
Soflex Discs (flexible discs) 4-step application	Aluminum oxide discs -coarse (50 µm) -medium (40 µm) -fine ( 24 µm) -extrafine (8 µm)	3 M ESPE (St. Paul, MN, USA) 4,673,856
Astropol assortment (rubber) 3-step application	Astropol F (36.5 µm) Astropol P (12.8 µm) Silicon rubber, silicon carbide Astropol HP (3.5 µm) Silicon rubber, diamond particles, aluminium oxide titanium oxide, iron oxide	Ivoclar Vivadent (Schaan,Liechtenstein) YL0369
Charisma Easyshine 1-step application	Microfine diamond powder and silicon carbide in a polyurethane matrix (5 µm)	Heraeus Kulzer (Hanau, Germany) 444,288

### Sample size calculation

Sample size calculation was performed using www.openepi.com. A minimal sample size of 7 in each subgroup was needed to detect a difference with a power of 95% at 95% confidence level.

### Sample preparation

A total of 224 composite resin samples were prepared, with 56 samples from each of the four composite resin types. Disc-shaped samples with a diameters and thicknesses of 8 and 2 mm, respectively, were prepared using a customized Teflon mold. A thickness of 2 mm was selected to ensure the opacity of the discs, regardless of the optical properties of the resin composite [16, 17]. An 8 mm diameter disc was selected to ensure accurate color reading and analysis using the tip of the spectrophotometer. The mold was placed on a flat surface, and the composite was packed using an Optrasculpt pad. The top surface of the sample was covered with a Mylar strip to ensure a smooth flat top surface and pressed with a glass slide to extrude excess material. A polywave-LED curing light (Bluephase style, Ivoclar, Vivadent) with energy density 1100 mW/cm^2^, was used to activate the composite resin for 40 s on each side. The samples of each composite resin were divided into four groups according to the polishing technique, with each group containing 14 specimens. The first group (no polishing) was considered a control and did not undergo any surface polishing. The second group (4-step) was polished using a four-step aluminum oxide disc (Soflex Discs). The third group (3-step) was polished using a three-step diamond silicon rubber disk (Astropol). The fourth group (1-step) was polished using a one-step polishing point (Charisma Easyshine). All polishing procedures were performed using a low-speed handpiece according to the manufacturer’s instructions.

For the Soflex discs, specimens were polished with coarse and medium discs at 10,000 rpm, then with fine and extrafine discs at 30,000 rpm, using light pressure. Each step involved repetitive circular strokes for 20 s, with efforts to standardize force and stroke number and each disc was discarded after one use. Astropol was used at 10,000 rpm with a slight rotary movement for 20 s. Easy-shine polishers were also used at the same speed, with 20 s of medium pressure followed by 20 s of light pressure. Abrasive discs were replaced after every three specimens. All polishing was done by the same operator to avoid variability,

### Storage media

To compare the ability of different materials to resist discoloration, half the samples were stored in distilled water, and half were stored in coffee staining solution (*n* = 7). The coffee staining solution was prepared by dissolving 2.4 g of coffee in 200 ml of boiling water and stirring for 10 min, followed by filtration through coffee filter paper. Samples were stored in coffee for 12 days to simulate a year of drinking coffee [18]. All groups were incubated at 37^◦^C for 12 days. The immersion solution was changed every day. After 12 days of immersion, the specimens were thoroughly rinsed with distilled water for 5 min and dried with tissue paper before color measurement.

### Color measurements

A Vita Easy shade guide spectrophotometer (Vita Zahn Fabric, Bad Sackingen, Germany) was used for color assessment using the Commission Internationale d’Eclairage L*a*b* coordination. The spectrophotometer was calibrated before each measurement according to the manufacturer's instructions. For each sample, color measurements were recorded three times by the same operator to ensure correct measurements. The spectrophotometer shade guide was connected to the Vita mobile assist application via Bluetooth, to record a, b, and L values. The same background was used for color measurements. The first color assessment was performed 24 h after polymerization and polishing to ensure complete polymerization [19, 20]. The second color measurement was recorded after 12 days of storage.

Color variation was calculated from the following equation:

$$\Delta E=\surd {([(\Delta L)}^{2}+ {(\Delta a)}^{2}+ {(\Delta b)}^{2}])$$

Here, L* is the lightness of the color and ranges from 0 to 100 (0 is the darkest and 100 is the lightest), a* is the color on the green–red axis (+ a* is red and -a* is green), and b* is the color on the blue-yellow axis (+ b* is yellow and -b* is blue). An ∆E < 2.7 was considered clinically acceptable in the present study [12]. Color measurements of the experimental groups immersed in water and coffee were also recorded.

### Scanning electron microscopic examination

After polishing, one representative specimen from each group was selected for scanning electron microscopic evaluation. Scanning electron microscopy (SEM) images were obtained from the most representative area using an environmental scanning microscope (FESEM; Quanta FEG 250, Netherlands) at a working voltage of 30 kV, and no sample preparation was required. Micrographs of each surface were captured at × 500 to further inspect the microstructural changes after polishing.

### Statistical analysis

The data are presented as mean, standard deviation (SD), minimum, and maximum, when appropriate. Normality of the data were analyzed using the Anderson–Darling test. As ΔE showed normal distribution, three-way analysis of variance ANOVA was used to compare the tested groups and control followed by Tukey HSD for pairwise comparison. The significance level was set at ^*P*^< 0.05. Statistical analysis was performed using IBM® SPSS® (ver. 26. SPSS Inc., IBM Corporation, Armonk, NY, USA).

## Results

The three-way ANOVA revealed that the material type, polishing techniques, storage media, and interaction between them all were significant factors influencing the ∆E values (*P* < 0.001) (Table 3).

**Table 3:** Three-way ANOVA results of ∆E

Source	Type III Sum Squares	df	Mean Square	F	Sig
**Materials**	714.277	3	238.092	963.594	< 0.001*
**Polishing**	5179.718	3	1726.573	6987.693	< 0.001*
**Media**	8115.863	1	8115.863	32,846.093	< 0.001*
Materials * Polishing * Media	205.163	9	22.796	92.258	< 0.001*
**Error**	47.441	192	0.247		
**Total**	32,838.819	224			
**Corrected Total**	20,573.597	223			

^*^ significant, *NS* non-significant

### Effect of material types on color change (ΔE)

Regardless of the polishing technique and storage medium, different material types showed a significant color change (ΔE) at ^*P*^ < 0.001. Filtek exhibited the most significant color change, followed by Gradia and Palfique, with no significant differences between them. Injectable formulation exhibited the lowest color change (Table 4). The color change for all composite types was above the clinically acceptable range (> 2.7).

**Table 4:** Mean and standard deviation (SD) results of ΔE for different tested materials

	**Gradia**		**Injectable**		**Filtek**		**Palfique**		^***P***^** -value**
	Mean	SD	Mean	SD	Mean	SD	Mean	SD	
ΔE	7.58b	9.28	4.81c	5.96	9.85a	12.64	7.36b	8.93	< 0.001*

Different letters within each row indicates significant difference at ^*p*^ < .05

^*^ significant, *NS* non-significant

### Effect of polishing technique on color change (ΔE)

Results of ΔE for the different polishing techniques tested, regardless of other variables, are presented in Table 5.

**Table 5:** Mean and standard deviation (SD) results of ΔE for different tested polishing techniques

	No Polish		4-Steps		3-Steps		1-Step		^*P*^ -value
	Mean	SD	Mean	SD	Mean	SD	Mean	SD	
ΔE	15.71a	15.32	4.85b	3.86	4.06c	3.34	4.98b	4.37	< 0.001*

Different letters within each row indicates significant difference at ^*p*^ < .05

^*^ significant, *NS* non-significant

Different polishing techniques resulted in significant color changes (^*P*^ < 0.001). The highest degree of color change was seen in the no-polishing group, followed by the 4-step and 1-step polishing groups, with negligible differences between each other. The lowest color change was observed in the 3-step polishing group, which was significantly lower than that observed in all other polishing groups. However, ΔE was more than the clinically acceptable range (> 2.7) for all groups.

### Effect of storage media on color change (ΔE)

Storage media had a significant effect on ΔE values at ^*P*^ < 0.001, regardless of the other variables as presented in Table 6. The water exhibited the lowest ΔE compared to coffee. The ΔE was below the clinically acceptable range (ΔE < 2.7) for water. This color change was above the clinically acceptable range for coffee (ΔE > 2.7).

**Table 6:** Mean and standard deviation (SD) results of ΔE for different tested storage media

	Water		Coffee		^*P*^ -value
	Mean	SD	Mean	SD	
ΔE	1.38	0.7	13.42	10.57	< 0.001*

Different letters within each row indicates significant difference at ^*p*^ < .05

^*^ significant, *NS* non-significant

### Comparison of ΔE among different polishing techniques with respect to material type and storage media

**Fig. 1:**
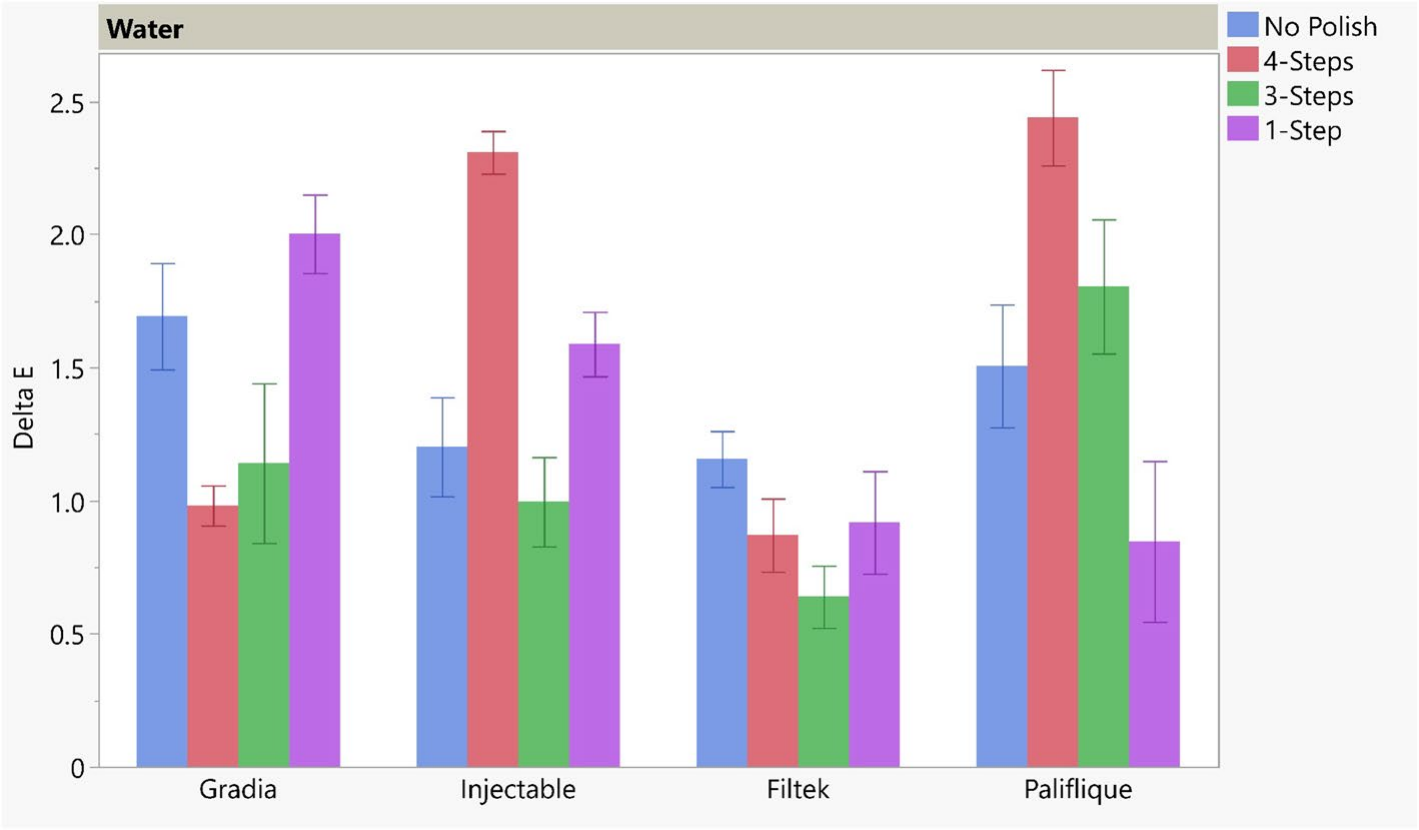
Bar showing mean ΔE for different polishing techniques for water storage

**Fig. 2:**
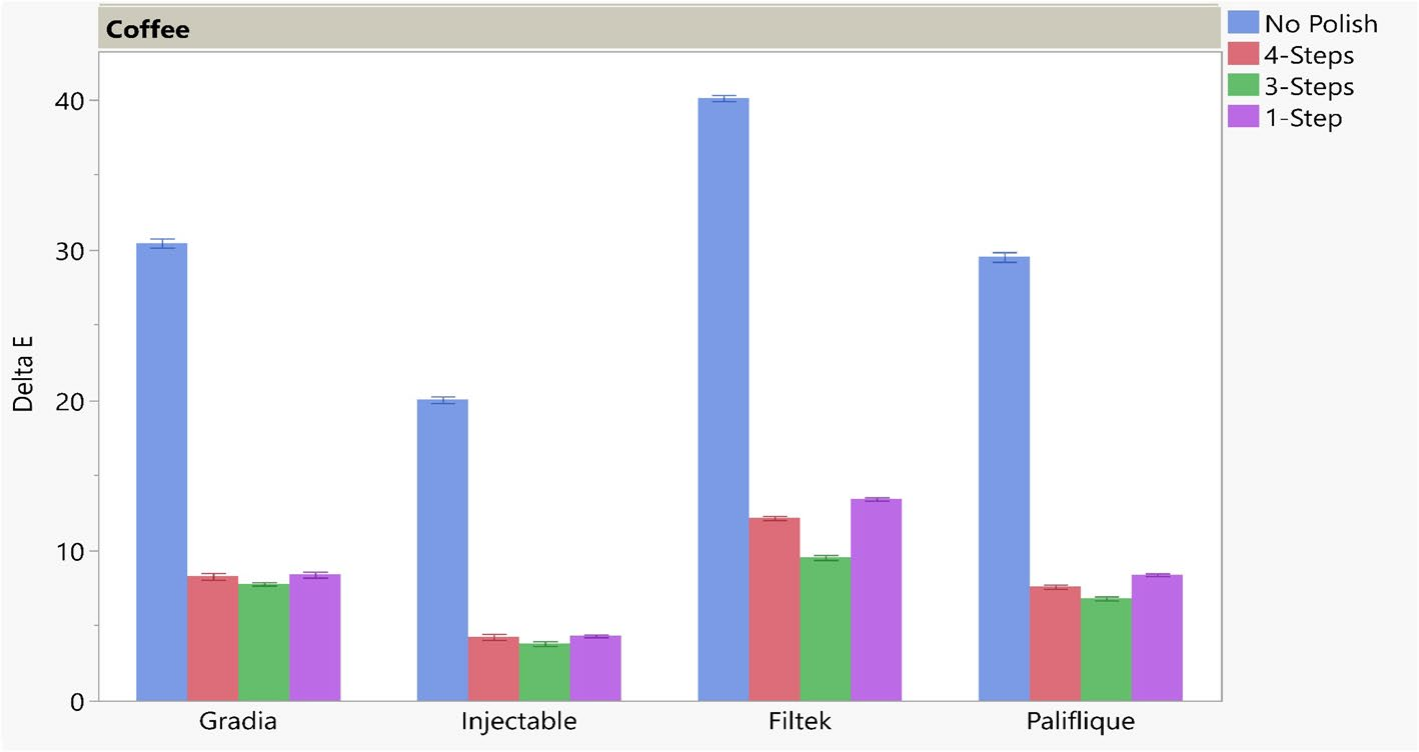
Bar showing mean ΔE for different polishing techniques tested for coffee storage

The results of ΔE for the different polishing techniques tested for different material types in water and coffee are presented in Table 7 and Figs. 1 and 2.

**Table 7:** Mean, Standard deviation (SD), and standard error results of ΔE for different tested polishing tech

		**No Polish**			**4-Steps**			**3-Steps**			**1-Step**			***P* -value**
		Mean	SD	SE	Mean	SD	SE	Mean	SD	SE	Mean	SD	SE	
Water	Gradia	1.69a	0.53	0.19	0.98b	0.20	0.07	1.14ab	0.79	0.30	2.00ab	0.39	1.89	< 0.001*
	Injectable	1.20a	0.49	0.19	2.31b	0.21	0.08	1.00a	0.44	0.17	1.59a	0.32	0.12	< 0.001*
	Filtek	1.16a	0.28	0.11	0.87a	0.36	0.14	0.64a	0.31	0.12	0.92a	0.51	0.19	0.284 NS
	Palifique	1.51a	0.61	0.23	2.44b	0.48	0.18	1.80b	0.67	0.25	0.85a	0.80	0.06	< 0.001*
Coffee	Gradia	30.44a	0.81	0.30	8.27b	0.60	0.23	7.77b	0.29	0.11	8.39b	0.53	8.27	< 0.001*
	Injectable	20.04a	0.60	0.23	4.23b	0.53	0.20	3.79b	0.41	0.16	4.30b	0.24	0.09	< 0.001*
	Filtek	40.10a	0.56	0.21	12.15c	0.35	0.13	9.53d	0.43	0.16	13.43b	0.29	0.11	< 0.001*
	Palifique	29.52a	0.85	0.32	7.58c	0.36	0.14	6.80d	0.35	0.09	8.38b	0.24	0.09	< 0.001*

Different letters within each row indicates significant difference at ^*p*^ < .05

^*^ significant, *NS* non-significant

In water storage, different material types showed significant color changes with the different polishing techniques, except for the Filtek group, which showed no significant difference (^*P*^ = 0.284). For Gradia, the lowest significant color change was with the 4-step polishing technique, followed by the 3-step and 1-step polishing techniques, which showed insignificant color changes between them. The most significant color change occurred without polishing. For the injectable group, 4-step showed the highest significant color change compared with all other polishing techniques, which presented insignificant differences. For the Palfique group, the no polish and 1-step polishing techniques showed significantly lower color change than 4-step and 3-step polishing. ΔE of all groups stored in water was lower than the clinical acceptable range (ΔE < 2.7).

During coffee storage, different material types showed significant color changes depending on the different polishing techniques. For the direct and injectable Gradia groups, the no-polish group showed the highest color change among all the polishing techniques. All the other polishing groups exhibited lower color changes, with insignificant differences. For the Filtek and Palfique groups, there was a significant color change among all the polishing techniques. The no-polish group exhibited the highest color change followed by 1-step, 4-step, and then 3-step polishing techniques. ΔE of all groups stored in coffee was higher than the clinical acceptable range (ΔE > 2.7).

### SEM micrographic evaluation

**Fig. 3:**
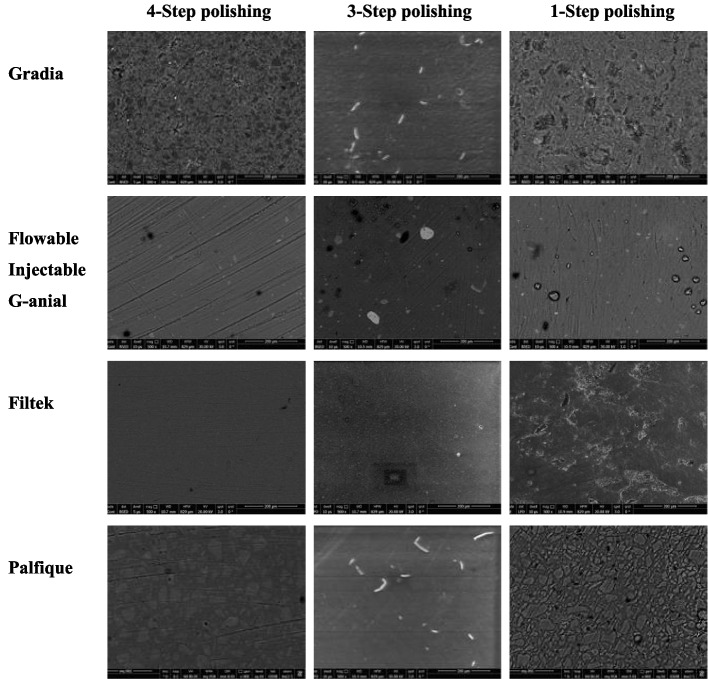
SEM micrographs at 500 × of the four composite resin types and polishing techniques

Figure 3 shows the SEM micrographs of the four composite resin types with different polishing techniques at 500 × magnification. Regardless of the polishing technique used, SEM micrographs of the nanocomposite resin types (Injectable, Filtek, and Palfique) with smaller filler particles revealed a smoother surface with fewer scratches and filler protrusions than the microhybrid composite (Gradia). SEM micrographs of the groups of 3-step technique revealed smoother surfaces for all composite types. The surface irregularities and pores were significant in the 1-step technique.

## Discussion

The study investigated the color stability of bleach-shaded composite resins, aiming to determine the most color stable type over time. Four resin types with varying components and filler particles were selected for the study.

The finishing and polishing procedures were intended to create smooth, shiny composite restorations with good contours and occlusion [14]. A smooth surface is also important to prevent plaque formation and bacterial adhesion onto composite resins and to decrease the possibility of secondary caries formation [8, 21]. Composite resin restorations are polished using various polishing techniques. However, it is unclear in the literature which technique produces a smoother surface. Additionally, the variation in filler size, type, and loading across different types of composite resins may require the use of a specific polishing protocol with a specific type of composite [15]. Four finishing and polishing protocols were used for comparison.

There have been reports of discoloration at three levels: the surface, subsurface, and body. The most prevalent cause of surface and subsurface staining of composite resins is the increased intake of stained beverages and foods [6, 7]. Coffee is one of the most commonly consumed beverages worldwide and more significantly, it is highly consumed in our society. Previous research has demonstrated that coffee contain stronger chromogens and yellow pigments of small molecular weight as compared to tea or cola, which lead to considerable amounts of staining on resin components [9]. Therefore, coffee was selected for the present study.

The colors of all samples were recorded using a reflective spectrophotometer because it is often impossible, ineffective, and unpredictable to quantitatively evaluate color changes through visual assessment. Spectrophotometers are considered the most accurate and reliable tools for clinical assessments, especially when compared with visual assessments [22]. In addition, as electronic optics and informatics advance, electronic techniques for color selection have become more affordable for everyday use [22]. Furthermore, these systems are more accurate than calorimeter measurements when environmental luminosity is considered [23].

This study uses the CIE L* a* b* coordinates to describe the color and provides information about the location of the color of the object in a uniform three-dimensional color space. Under controlled circumstances, 50% of observers can visually detect a color difference of about one ΔE unit. The literature reports a wide range of cutoffs for clinically acceptable ΔE levels. In this study, ΔE values under 2.7 were considered clinically acceptable [24].

The results of our study revealed substantial color change depending upon material type, polishing technique, storage media, and their interaction, resulting in a significant effect on ΔE. Therefore, the null hypothesis was rejected. The variable behavior of different materials with respect to color change may be related to the composition and nature of the components of the resin material. Finer particle size results in reduced interparticle spacing, filler plucking, better polishing outcomes, and color change [25]. Upon examining the material compositions, it was observed that not only the filler size and type but also the resin matrix plays a role. The injectable nanohybrid exhibited greater resistance to discoloration; its resin matrix consisted of Bis-MEPP, Bis-EMA UDMA, and TEDMA. While the material that exhibited the greatest color change was the Filtek nanofill, its resin matrix was Bis-GMA and TEGDMA. The presence of more hydrophilic co-monomers could be responsible for the discoloration due to increased susceptibility to water absorption and other coloring agents [26]. Additionally, composite resins containing TEGDMA in their composition release significantly more monomers in aqueous media than composite resins based on UDMA, leading to a greater change in color [27]. According to one study, urethane dimethacrylate is more stain-resistant than bisphenol A glycidyl dimethacrylate [28].

Kheraif and co-authors discovered that nanoresin composite showed much higher discoloration than microhybrid composite, in line with the aforementioned results [29]. Other investigations have reported that microhybrid composites are more susceptible to external discoloration than nanohybrid composites because of the insufficient polishing capacity of the microfillers [7, 20].

After immersion in coffee, a comparison of the color stability of the different materials used in the study revealed that the results were more dependent on the polishing methods and did not follow a consistent pattern.

With respect to the polishing techniques, the Mylar strip (no polishing) showed the most significant color change compared to the other polishing systems. Using a Mylar strip without polishing leaves an oxygen-inhibited layer, resulting in a polymer-rich surface that makes the restoration relatively unstable and easily discolorable [13]. Furthermore, this surface should be removed because its high resin content can make it prone to wear in an oral environment. If no polishing technique is used, the oral environment is exposed to inorganic fillers. The effectiveness of finishing and polishing techniques is crucial to the clinical success of composite resin restorations because this layer is frequently abolished during the removal of extra material or contouring of the restoration after implantation [30].

The cutting particles must be coarser than the filler particles for an effective finishing system. Otherwise, polishing will only remove the soft resin matrix, leaving the filler particles sticking out from the surface, increasing the surface roughness and ultimately leading to a color change [31].

The aim of this study was to determine whether a particular polishing technique can be recommended for a certain composite type. Our results revealed that the three-step polishing technique using silicone rubber discs containing diamond particles (Astropol) showed the lowest color change for all composite types, followed by the 4-step disc technique (Sof-Lex), while the highest change in color was observed with the one-step technique (Chairsma Easyshine). A recent systematic review suggested that the most effective polishing systems are those in which the abrasive particle size is systematically reduced, as in the case with the 3-step Astropol system and 4-step Sof-Lex Discs system [32]. The Sof-Lex disc's aluminum oxide cutting particles are tougher than the Astopol's silicon cutting particles and filler particles of the resin composite used. This property enables the removal of both fillers and soft resin matrix during finishing [33]. Moreover, several studies have concluded that the three-step finishing protocol is superior to the one-step technique, supporting the above results [7, 20, 22]. Another study reported that a three-step polishing technique produced the highest gloss values, followed by a two-step polishing system, while a one-step polishing system produced the lowest gloss values [34].

However, there are discrepancies in the literature regarding the most effective finishing process for composite resins. According to Schmitt and co-authors, a nanofilled composite outperformed a microhybrid composite prepared with a three-step finishing and polishing procedure in terms of color stability [35]. Another study observed better color stability in the Sof-Lex polishing group, followed by the Astropol polishing group [36]. Another recent study found that Sof-Lex exhibited better polishing and greater color stability than Astropol [37]. This variation in the results could be related to the differences in the composite types tested. These studies used Sof-Lex Discs as a finishing and polishing system in 3-steps, ignoring the first disk in the system stream, whereas in our study, we used it in four steps.

Tian et al. (2012) indicated that distilled water could be used as a control because prior experiments documented minor color changes in composite resins [38]. In this study, the groups stored in water did not show any significant color change among all the composite types, whether polished or unpolished. In addition, none of the tested materials had ΔE values greater than 3.3. This may indicate that the bleach-shaded composite resins are relatively color-stable, provided that the material is not subjected to any coloring agent. Additionally, dental composites have low solubility in water, and the process of water sorption is slow.

During coffee storage, all groups, whether polished or unpolished, showed significant color changes that were clinically visual to the human eye. Coffee is a potent chromogen, and because coffee pigments have low molecular weights, they can quickly saturate resin composites. The affinity between the polymer phase and yellow colorants in coffee is responsible for the absorption and penetration of the colorants into the organic phase of the resin-based materials. This phenomenon could be attributed to various staining compounds present in coffee, including gallic acid, which further exacerbates its staining potential [39].

In addition to color stability, the surface topography of resin-based materials is an important factor affecting the clinical success of esthetic restorations. Representative SEM micrographs were interpreted to observe the effects of the different polishing techniques on the different components of the composite resin. SEM analysis showed that the composites with small spherical filler particles had smoother surfaces and fewer scratches. Unlike microhybrid composites with larger filler particles, less material was removed from the surface of the nanocomposite during polishing because of the stronger interparticle adhesion of nanotechnology-produced particles. The three-step polishing yielded a more homogeneous surface for most composite types. However, some studies have indicated that the performance of a particular polishing system depends on the type of composite resin and that the surface roughness of composite is more closely related to the material composition than the polishing system employed [40–43].

One of the limitations of the present study is that it is an in vitro test that provides an overview of the material behavior. Other factors may have contributed to these clinical results. Further research is needed to better understand how these resins and polishing systems perform in clinical situations involving the complex oral environment. The limited sample size is considered another limitation, although we employed advanced statistical analyses to ensure the reliability of our results, larger sample sizes are recommended for validation and extension of our findings.

## Conclusions

Thus, the null hypothesis was rejected. Considering the limitations of this study, the following conclusions were drawn:

1. Proper finishing and polishing procedures improve color stability of the bleach-shaded composite resins.
2. The 3-step polishing system with silicone rubber discs containing diamond particles exhibited the lowest color change among all tested composite resins and is likely to improve clinical success.
3. Coffee has a deleterious effect on the color of bleach-shaded composite resins and can cause significant color changes over time.
4. Injectable flowable nanohybrid composites appeared to be more resistant to coffee staining than the other tested composite resins.

## Recommendations

Further studies are required to find out how to improve the staining susceptibility of the bleach-shaded composites highlighting the intrinsic factors and material composition. More comprehensive studies are required to study the surface topography of polished bleach shaded composite resin of different filler compositions. Moreover, A special attention shall be given to properties of injectable composite resin as well as durability. Finally, clinical investigations are necessary to validate the results of this study.

## Data Availability

The datasets supporting the conclusions of this article are included within the article and its additional files.
